# Toripalimab plus chemotherapy as second-line treatment in previously EGFR-TKI treated patients with EGFR-mutant-advanced NSCLC: a multicenter phase-II trial

**DOI:** 10.1038/s41392-021-00751-9

**Published:** 2021-10-15

**Authors:** Tao Jiang, Pingyang Wang, Jie Zhang, Yanqiu Zhao, Jianying Zhou, Yun Fan, Yongqian Shu, Xiaoqing Liu, Helong Zhang, Jianxing He, Guanghui Gao, Xiaoqian Mu, Zhang Bao, Yanjun Xu, Renhua Guo, Hong Wang, Lin Deng, Ningqiang Ma, Yalei Zhang, Hui Feng, Sheng Yao, Jiarui Wu, Luonan Chen, Caicun Zhou, Shengxiang Ren

**Affiliations:** 1 Shanghai Pulmonary Hospital, Tongji University School of Medicine, Shanghai, China; 2 State Key Laboratory of Cell Biology, Innovation Center for Cell Signaling Network, CAS Center for Excellence in Molecular Cell Science, Shanghai Institute of Biochemistry and Cell Biology, Chinese Academy of Sciences, University of Chinese Academy of Sciences, Shanghai, China; 3 Henan Cancer Hospital, Zhengzhou, China; 4 The First Affiliated Hospital of Zhejiang University, Hangzhou, China; 5 Zhejiang Cancer Hospital, Hangzhou, China; 6 Jiangsu Province Hospital, Nanjing, China; 7 The Fifth Medical Center of PLA General Hospital, Beijing, China; 8 Tangdu Hospital, Fourth Military Medical University, Xi’an, China; 9 The First Affiliated Hospital of Guangzhou Medical University, Guangzhou, China; 10 Shanghai Junshi Biosciences Co. Ltd, Shanghai, China; 11 Key Laboratory of Systems Health Science of Zhejiang Province, Hangzhou Institute for Advanced Study, University of Chinese Academy of Sciences, Hangzhou, China; 12 School of Life Science and Technology, ShanghaiTech University, Shanghai, China

**Keywords:** Predictive medicine, Clinical trials, Lung cancer, Immunotherapy

## Abstract

This multicenter phase-II trial aimed to investigate the efficacy, safety, and predictive biomarkers of toripalimab plus chemotherapy as second-line treatment in patients with *EGFR*-mutant-advanced NSCLC. Patients who failed from first-line EGFR-TKIs and did not harbor T790M mutation were enrolled. Toripalimab plus carboplatin and pemetrexed were administrated every three weeks for up to six cycles, followed by the maintenance of toripalimab and pemetrexed. The primary endpoint was objective-response rate (ORR). Integrated biomarker analysis of PD-L1 expression, tumor mutational burden (TMB), CD8 + tumor-infiltrating lymphocyte (TIL) density, whole-exome, and transcriptome sequencing on tumor biopsies were also conducted. Forty patients were enrolled with an overall ORR of 50.0% and disease-control rate (DCR) of 87.5%. The median progression free survival (PFS) and overall survival were 7.0 and 23.5 months, respectively. The most common treatment-related adverse effects were leukopenia, neutropenia, anemia, ALT/AST elevation, and nausea. Biomarker analysis showed that none of PD-L1 expression, TMB level, and CD8 + TIL density could serve as a predictive biomarker. Integrated analysis of whole-exome and transcriptome sequencing data revealed that patients with *DSPP* mutation had a decreased M2 macrophage infiltration and associated with longer PFS than those of wild type. Toripalimab plus chemotherapy showed a promising anti-tumor activity with acceptable safety profiles as the second-line setting in patients with *EGFR*-mutant NSCLC. *DSPP* mutation might serve as a potential biomarker for this combination. A phase-III trial to compare toripalimab versus placebo in combination with chemotherapy in this setting is ongoing (NCT03924050).

## Introduction

The therapeutic landscape has dramatically shifted during the past decade for advanced non-small-cell lung cancer (NSCLC) patients with *EGFR*-sensitizing mutations.^1,2^ First-line setting with EGFR-TKIs, such as erlotinib, gefitinib, icotinib, afatinib, dacomitinib, and osimertinib, has resulted in significantly longer progression-free survival (PFS) than the previous standard of care.^3–9^ However, after failure of first-line EGFR-TKI therapy, PFS with second or subsequent lines of treatment is disappointing with 4.4–5.4 months in *EGFR* T790M-negative group that received chemotherapy.^10–12^ Even though those with *EGFR* T790M mutations received osimertinib, resistance is also inevitable and subsequent treatments often show very limited clinical efficacy.^1,13^ Thus, a novel strategy is urgently needed to further improve the prognosis of these patients after failure to *EGFR*-TKIs.

Currently, immune-checkpoint inhibitors (ICIs) targeting the PD-1/PD-L1 interactions have shifted the therapeutical landscape of advanced/metastatic NSCLC without driver (e.g., *EGFR*, *ALK*, and *ROS1*) mutations. It has been incorporated into standard of care as a second-line treatment in advanced NSCLC.^14–17^ In addition, pembrolizumab or atezolizumab monotherapy is also approved as the first-line treatment in advanced NSCLC patients who have high PD-L1 expression in tumor tissues.^18,19^ However, anti-PD-(L)1 monotherapy did not show substantial survival benefits than standard chemotherapy in those who failed to *EGFR*-TKIs.^20–22^ Furthermore, clinical trials of osimertinib plus PD-1 blockade resulted in high rate of interstitial pneumonitis,^23,24^ suggesting that an alternative strategy is needed in this setting.

Comprehensive depiction of the tumor microenvironment (TME) might help clarify the mechanism of lower response and guide the subsequent therapeutic strategy. Previously, we and others have found that *EGFR*-mutant lung cancers are more likely to have lower PD-L1 expression, fewer CD8 + tumor-infiltrating lymphocytes (TILs), and lower tumor mutational burden (TMB) level than *EGFR* wild-type tumors.^20,25^ Additional evidence showed that chemotherapy-induced neoantigen release could modulate the TME to have a potentially synergistic effect with ICIs.^26,27^ Meanwhile, the addition of atezolizumab to bevacizumab plus chemotherapy showed superior efficacy in patients with *EGFR*-mutant NSCLC from IMPOWER 150 study,^28^ indicating potent clinical benefit of chemoimmunotherapy combination in this setting.

Toripalimab, a humanized IgG4κ mAb with hinge mutation (S228P) specific for human PD-1 receptor and blocks interactions of PD-1/PD-L1, has shown an acceptable safety profile and promising clinical activities in patients with advanced solid tumors in several phase-I/II studies^29–31^ and has been approved as the second-line setting for metastatic melanoma in 2018, China. Currently, several ongoing studies are investigating the combination of toripalimab with standard-of-care regimens as the first-line treatment in patients with NSCLC.

In order to evaluate the efficacy and safety of toripalimab plus carboplatin and pemetrexed in previously *EGFR*-TKI-treated patients with *EGFR*-mutant NSCLC, we conducted this open-label, single-arm phase-II trial and enrolled 40 patients from eight medical centers in China. We further performed integrated analysis of multi-omic data from PD-L1 expression, TMB, CD8 + TIL density, whole-exome, and transcriptome sequencing on tumor biopsies to identify the potential predictive biomarkers.

## Results

### Patient population

**Fig. 1:**
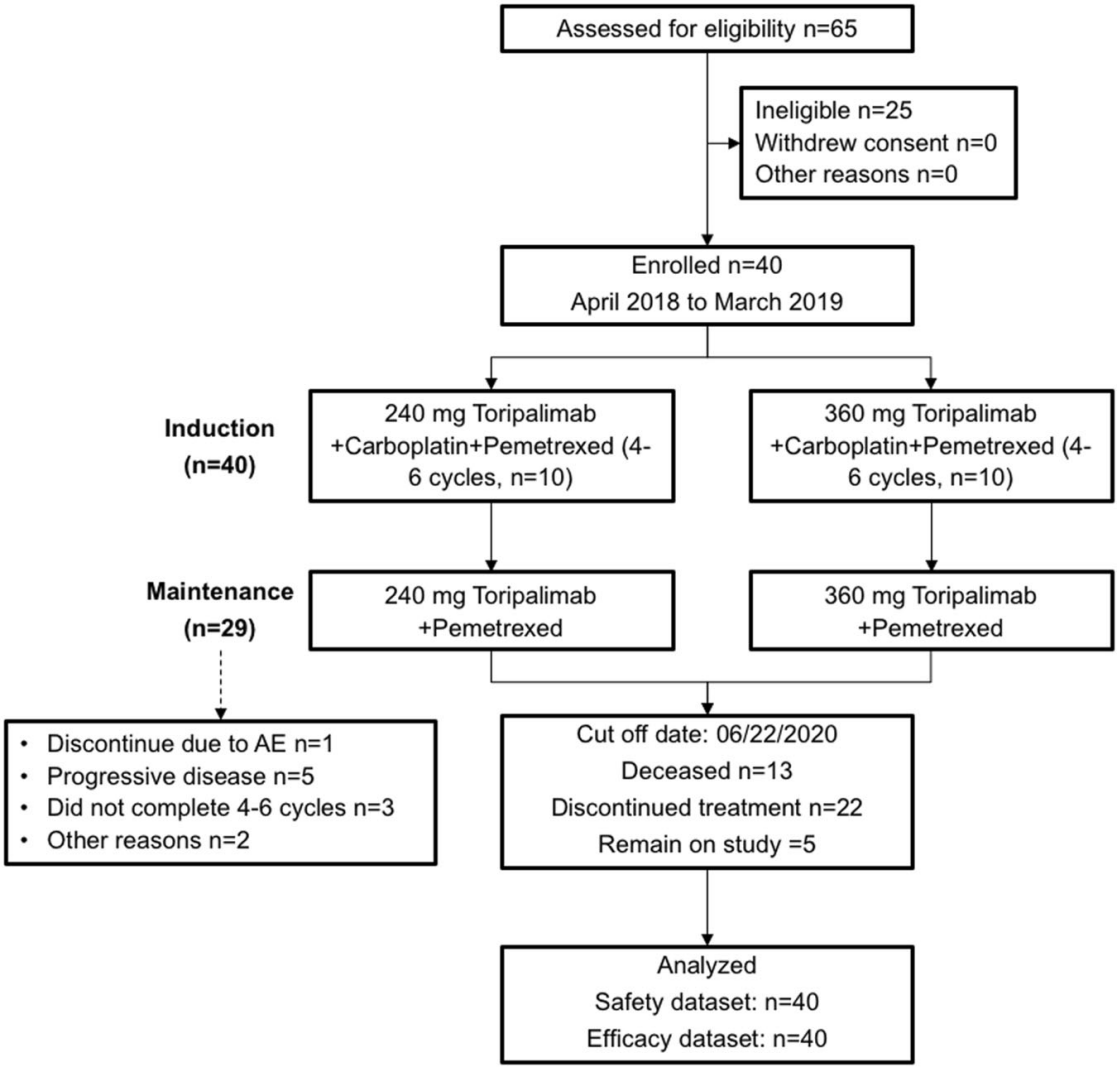
CONSORT diagram for this phase-II study

From April 2018 to March 2019, 40 patients were enrolled (Fig. 1). Baseline characteristics are summarized in Table 1. The median age was 58 years (range: 19–73), including 21 (52.5%) female and 19 (47.5%) male patients. Twenty-three (57.5%) patients had *EGFR* Exon 19 deletion (*EGFR* 19DEL) and 17 (42.5%) patients harbored Exon 21 L858R mutation (*EGFR* L858R). Twenty (50.0%) patients received gefitinib as first-line treatment, whereas 16 (40.0%) received icotinib and 4 (10.0%) received erlotinib. All the enrolled patients did not develop *EGFR* T790M mutation in the rebiopsy. Thirty patients received 360-mg dose of toripalimab treatment and 10 patients received the 240-mg dose.

**Table 1:** Summary of patient demographics

Characteristics	Patients (*N* = 40) *n* (%)
Sex: male/ female	19 (47.5)/ 21 (52.5)
Age, median (range), years	57.5 (19–73)
Race: Asian	40 (100)
ECOG PS: 0/ 1	2 (5)/ 38 (95)
Smoking history: yes/ no	10 (25)/ 30 (75)
EGFR mutations: Ex19del/ L858R	23 (57.5)/ 17 (42.5)
CNS metastases	6 (15)
Previous EGFR-TKI: gefitinib/erlotinib/icotinib	20 (50)/ 4(10)/ 16(40)
PD-L1 tumor proportion score**:	
Negative	19 (47.5)
Positive	21 (52.5)

ECOG Eastern Cooperative Oncology Group, TNM Tumor, node, metastasis staging system, LDH Lactate dehydrogenase

**Positive defined as ≥1% of tumor cells expressing PD-L1 by JS311 IHC staininge

### Efficacy

**Fig. 2:**
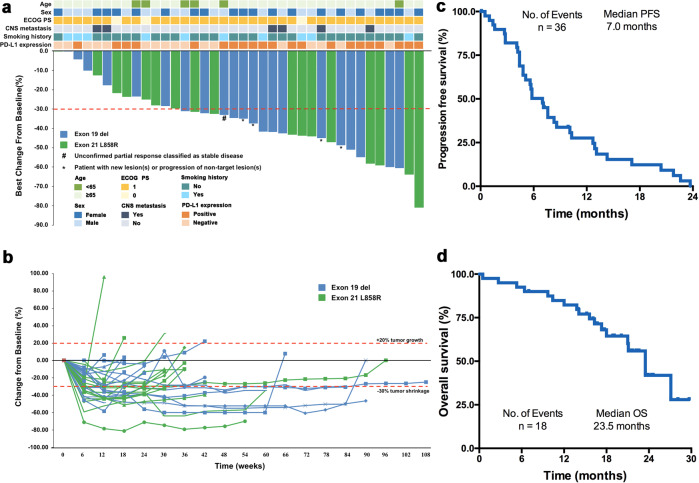
Summary of treatment response, progression-free and overall survival. **a** Maximal change of tumor size from baseline assessed by the investigator per RECIST v1.1 (*n* = 38). The length of the bar represents maximal decrease or minimal increase in target lesion(s). # Unconfirmed partial response classified as stable disease. * Patient with target lesion(s) reduction over 30% but with new lesion(s) or progression of nontarget lesion(s). **b** Change of individual tumor burden over time from baseline assessed by the investigator per RECIST v1.1 (*n* = 38). **c** Progression-free survival by RECIST v1.1 of all 40 enrolled patients. **d** Overall survival by RECIST v1.1 of all 40 enrolled patients. No. number

As of October 22, 2020, the median follow-up time was 10.5 months. In total, 18 (45.0%) patients died, 22 (55.0%) discontinued treatment due to disease progression (PD), 3 (7.5%) discontinued due to adverse effects, and 2 (5.0%) patients remained on study. The overall confirmed objective-response rate (ORR) was 50.0% (95%CI 33.8–66.2), and the disease control rate (DCR) was 87.5% (95%CI 73.2–95.8). Tumor shrinkage was observed in 36 (90.0%) patients (Fig. 2a). The median duration of response (DOR) was 7.0 months (Fig. 2a), median PFS was 7.0 months (95%CI 4.8–8.4), and median overall survival (OS) was 23.5 (95% CI 18.0 to NR months) (Fig. 2c, d). Among patients who completed the induction treatment (*n* = 29), the ORR was 69.0%.

Subgroup analysis showed that the ORR was 58.8% in patients with *EGFR* L858R and 43.5% in patients with *EGFR* 19DEL (Supplementary Table S1). Patients with *EGFR* L858R had numerically longer median PFS (7.6 versus 5.4 months, *P* = 0.639) and OS (23.5 versus 18.0 months, *P* = 0.164) than patients with *EGFR* 19DEL (Supplementary Fig. S1a and S1b). Patients in 360 mg of toripalimab cohort (*n* = 30) and 240 mg of toripalimab cohort (*n* = 10) had similar ORR. Subgroup analysis of clinical characteristics, including age, gender, prior TKI treatment, and liver/central nervous system (CNS) metastasis, was seen in Supplementary Table S1. As to the subgroup analysis, we found that patients with LDH at normal range had numerically better ORR than those with LDH above the upper limit of normal (54.3% versus 20.0%, *P* = 0.34). In addition, three patients with liver metastasis had no objective response, whereas four out of six (66.7%) patients with CNS metastasis had partial response.

### Safety

As data cutoff, 39 out of 40 (97.5%) of patients experienced treatment-related adverse events (TRAE, Table 2). The most common (≥20%) TRAE included 33 (82.5%) leukopenia, 28 (70.0%) neutropenia, 27 (67.5%) anemia, 21 (52.5%) elevated AST, 20 (50.0%) elevated ALT, 19 (47.5%) nausea, 19 (47.5%) thrombocytopenia, 15 (37.5%) decreased appetite, 11 (27.5%) constipation, and 10 (25.0%) asthenia. Grade 3 and above TRAE occurred in 26 patients (65.0%), including 16 (40.0%) neutropenia, 9 (22.5%) leukopenia, 4 (10%) thrombocytopenia, 2 (5%) of each anemia, pneumonia, and abnormal hepatic function, and 1 (2.5%) of each upper respiratory-tract infection, decreased appetite, maculo-papular rash, bone marrow suppression, febrile neutropenia, interstitial lung disease, and thoracic hemorrhage. Permanent treatment discontinuation of toripalimab due to TRAE occurred in 4 (10%) patients, while 15 (37.5%) patients had toripalimab delayed due to TRAE. The immune-related AEs (irAEs) included 16 (40.0%) thyroiditis, 12 (30.0%) skin toxicity, 10 (25.0%) hepatitis, 5 (12.5%) nausea, 3 (7.5%) uveitis, 1 (2.5%) pneumonitis, 1 (2.5%) conjunctivitis, 1 (2.5%) anemia, and 1 (2.5%) leukopenia. Grade 3 and above irAEs only occurred in 2 patients (5.0%), including 1 (2.5%) skin toxicity and 1 (2.5%) pneumonitis (Table 2).

**Table 2:** Treatment-related and immune-related adverse events in this study

AE parameter	CTCAE all incidence (%)	Grade 3–5 incidence (%)
Number of patients with at least one TRAE	39 (97.5)	26 (65.0)
Leukopenia	33 (82.5)	9 (22.5)
Neutropenia	28 (70.0)	16 (40.0)
Anemia	27 (67.5)	2 (5.0)
Aspartate aminotransferase increased	21 (52.5)	2 (5.0)
Alanine aminotransferase increased	20 (50.0)	2 (5.0)
Nausea	19 (47.5)	0 (0.0)
Thrombocytopenia	19 (47.5)	4 (10.0)
Decreased appetite	15 (37.5)	1 (2.5)
Constipation	11 (27.5)	0 (0.0)
Asthenia	10 (25.0)	0 (0.0)
Vomiting	7 (17.5)	0 (0.0)
Rash	6 (15.0)	0 (0.0)
Urinary tract infection	5 (12.5)	0 (0.0)
Gamma-glutamyl transferase increased	4 (10.0)	0 (0.0)
Influenza like illness	4 (10.0)	0 (0.0)
Lung infection	4 (10.0)	1 (2.5)
Pyrexia	4 (10.0)	0 (0.0)
Upper respiratory tract infection	4 (10.0)	1 (2.5)
*Immune-related AE*		
Thyroiditis	16 (40.0)	0 (0.0)
Skin toxicity	12 (30.0)	1 (2.5)
Hepatitis	10 (25.0)	0 (0.0)
Nausea	5 (12.5)	0 (0.0)
Uveitis	3 (7.5)	0 (0.0)
Pneumonitis	1 (2.5)	1 (2.5)
Conjunctivitis	1 (2.5)	0 (0.0)
Anemia	1 (2.5)	0 (0.0)
Leukopenia	1 (2.5)	0 (0.0)

### Predictive role of PD-L1 expression, TMB, and CD8+TIL density

Tumor PD-L1 expression was tested in all 40 patients by using JS311 assay, which previously showed comparable results with 28-8, 22C3, and SP263 antibodies in NSCLC^30^. Patients of positive PD-L1 expression had numerically higher ORR (61.9% versus 36.8%, *P* = 0.204; Supplementary Table S1), longer median PFS (7.6 versus 5.8 months, *P* = 0.424; Supplementary Fig. S2a), and OS (NR versus 21.0, *P* = 0.052; Supplementary Fig. S2b) than negative PD-L1 expression. Among patients of PD-L1 expression ≥10%, the difference in ORR (75.0% versus 39.3%, *P* = 0.081) and PFS (median 8.1 versus 5.8 months, *P* = 0.697; Supplementary Fig. S2c) was more pronounced, while not in OS (median 21.1 versus 23.5 months, *P* = 0.424; Supplementary Fig. S2d). TMB was determined in 34 patients. Patients with high TMB (>median) had analogous ORR (57.1% versus 50.0%, *P* = 0.738), PFS (median 7.1 versus 5.1 months, *P* = 0.422; Supplementary Fig. S2e), and OS (median 23.5 versus 23.5 months, *P* = 0.541; Supplementary Fig. S2f) to those with low TMB (≤median). Intriguingly, patients with high CD8 + TIL density have numerically shorter PFS (median 5.8 versus 7.1 months, *P* = 0.726; Supplementary Fig. S2g) and OS (median 18.0 versus 27.1 months, *P* = 0.275; Supplementary Fig. S2h) than those with low CD8 + TIL density.

### Whole-exome sequencing

**Fig. 3:**
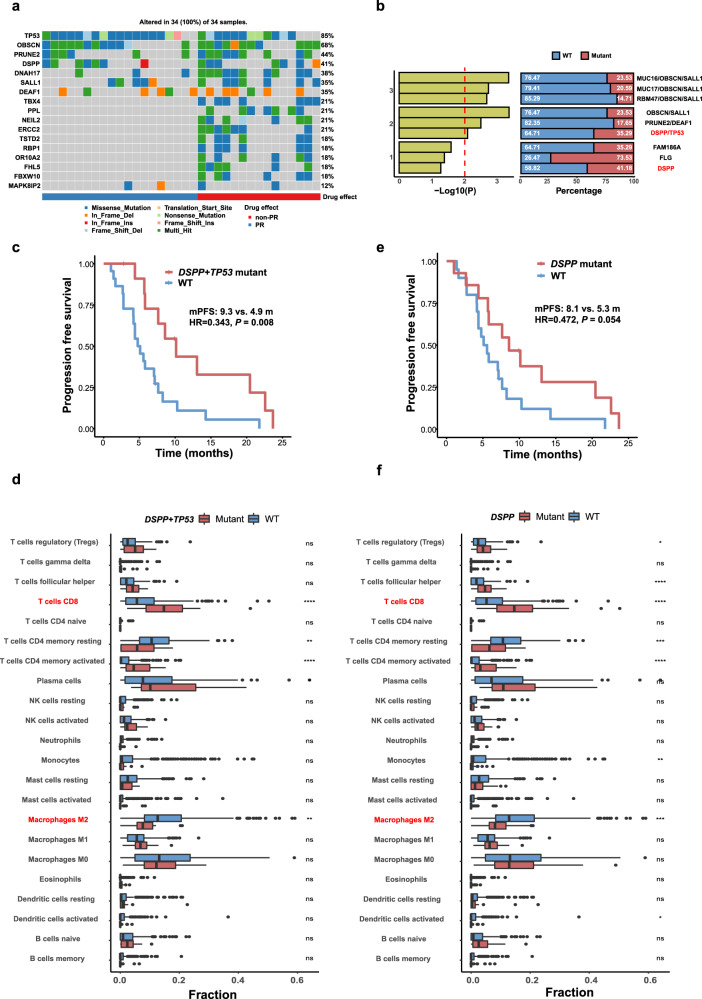
Whole-exome sequencing. **a** Highlight mutated genes color-coded by the type of mutations in experimental samples. **b** According to the *P* value of PFS between groups, the top three combinations with different gene numbers were selected. The red line in the left panel shows the threshold of the filter. The white number on the barplot in the right panel represents the proportion of the corresponding sample in the total population. **c** Progression-free survival in patients with *DSPP* + *TP53* comutation versus wild type (log-rank test). **d** Immune infiltration of *DSPP* + *TP53* comutation versus wild type from *EGFR-*mutant samples obtained from TCGA (Wilcoxon signed-rank test). **e** Progression-free survival in patients with *DSPP* mutation versus wild type (log-rank test). **f** Immune infiltration of *DSPP* mutation versus wild type from *EGFR* mutant samples obtained from TCGA (Wilcoxon signed-rank test). PFS progression free survival; HR hazard ratio; *P < .05, **P < .01, ***P < .001, ****P < .0001

Whole-exome sequencing (WES) was performed on both tumor biopsies and paired blood cells in 34 patients. WES identified 7048 genetic alternations, including 3505 missense mutations, 84 gene deletions, 123 rearrangements, 119 alternative splicing sites, 349 truncations, and 2748 gene amplifications. Having noticed the significant difference of gene alterations between patients with partial response (PR) versus non-PR (Fig. 3a), we inferred that genetic mutation might be utilized as predictive markers to this regimen. Prediction of genesets associated with efficacy was performed using Maftools::survGroup (v2.6.05) on the top 100 most frequently mutated genes.^32^ Considering the small size of data set, we set a relatively strict criteria (*P* ≤ 0.01) to screen genes or genes’ combination with predictive ability. We found that the prediction model will have an excellent effect when the number of components reaches two. In order to ensure the validity of our model, the minimum sample amount for each group should be more than 25% of the whole population (Fig. 3b).

The double mutations of *DSPP* and *TP53* (DT) were the most effective combination after screening, which were associated with significantly longer median PFS than wild type (9.3 versus 4.9 months, *P* = 0.008; Fig. 3c). Moreover, immune-infiltration analysis using CIBERSORTx in 354 *EGFR-*mutant patients from 18 projects of TCGA datasets showed that the DT double mutations have a significantly increased CD8^+^T cell and decreased M2 macrophage infiltration compared with wild-type tumors (Fig. 3d). Interestingly, *DSPP* could be used alone as a potential predictive biomarker (8.1 versus 5.3 months, *P* = 0.054; Fig. 3e), while *TP53* alone could not (Supplementary Fig. S3a). Compared with *TP53*, similarity in the characteristics of immune infiltration between *DSPP* mutation and DT double mutations was observed (Fig. 3f), suggesting the potential predictive value of *DSPP* mutation rather than *TP53* (Supplementary Fig. S3b). However, neither DT single nor double mutations cannot predict OS (Supplementary Fig. S3c–e).

### Whole transcriptome sequencing

**Fig. 4:**
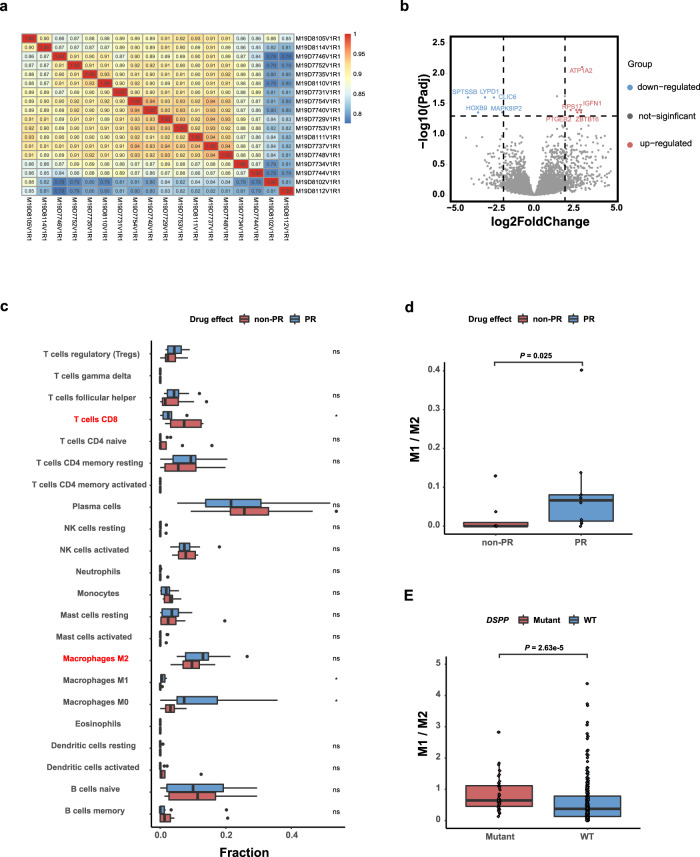
Integrated analysis of whole exome and transcriptome sequencing. **a** Sample correlation heat map based on expression matrix; different colors represent different correlation coefficients. **b** Volcano plot of eight upregulated and five downregulated differentially expressed genes between PR and non-PR group, each of them labeled the names of the first five genes. **c** Immune analysis of *PR* versus non-PR group from our cohort (Wilcoxon signed-rank test). **d** M1/M2 macrophage ratio of *PR* versus non-PR from our cohort (Wilcoxon signed-rank test). **e** M1/M2 macrophage ratio of *DSPP*-mutant versus wild-type tumors from *EGFR-*mutant samples obtained from TCGA (Wilcoxon signed-rank test). *P < 0.05, **P < 0.01, ***P < 0.001, ****P < 0.0001

To discover the differential gene expression and characteristics of immune infiltration corresponding to different therapeutic effects, we conducted RNA-seq on 18 patients with adequate tissue samples. In general, the similarity between samples is relatively strong (Fig. 4a). Only 13 differentially expressed genes (DEGs) were found between two groups (*P* ≤ 0.05, log_2_fold change ≥2), including eight upregulated and five downregulated genes (Fig. 4b). Moreover, we found that the expression value of these 13 genes could not be used to distinguish two different therapeutic groups directly. Considering the small number of DEGs and analogous expression value between two groups, we tried to use support vector machines (SVM) to select candidate marker genes and then identify genes with high-accuracy classification performance (Supplementary Fig. S4a). The results showed that six components in a set could have the precise distinguishing ability (Supplementary Fig. S4b). The gene set possesses the best grouping ability that consisted of six genes, including *RNF220*, *MAPK8IP2*, *PTGER2*, *P2RX4*, *ACADVL*, and *SPTSSB* (Supplementary Fig. S4c). After 1000 times independent random grouping experiments on our data set, we found that five of the six test samples can be grouped correctly 985 times with the predictor mentioned above, and 951 of them can be grouped into all pairs. Notably, differential expression analysis between PR and non-PR groups at the network level by sample-specific networks (SSN) also validated the grouping ability of the six-gene set (Supplementary Fig. S4c).

Then we focused on the analysis of distinct immune infiltration between PR and non-PR groups. We observed that patients with PR had significant lower CD8^+^T-cell abundance than those with non-PR (Fig. 4c). In addition, patients with PR had dramatically higher fractions of M1 macrophage than those with non-PR (Fig. 4c). Although there was no significant difference in the fraction of M2 macrophage between two groups, the ratio of M1/M2 was found to be markedly higher in PR group (*P* = 0.025; Fig. 4d), indicating the pivotal role of macrophage in determination of antitumor activity of PD-1 blockade plus chemotherapy in *EGFR*-mutant NSCLC.^33^

### Integrated analysis of whole exome and transcriptome sequencing

Having noticed the potential predictive significance of *DSPP* mutation, we performed the integrated analysis of whole-exome and transcriptome-sequencing data from the TCGA database to explicate the biological mechanism of the association between *DSPP* mutation and therapeutic effect. First, we conducted differential expression analysis and pathway enrichment. In total, 1612 DEGs were identified (1536 upregulated and 76 downregulated; *P* ≤ 0.05, log_2f_old change ≥2, Supplementary Fig. S5a). After sorting the Enrichment Score (ES) estimated by GSEA data, top 20 upregulated and downregulated pathways were selected. We found that mitogen-activated protein kinase (MAPK) signaling pathway gene set was significantly downregulated (ES = -0.309) in the *DSPP*-mutant group (Supplementary Fig. S5b). Protein–protein-interaction network (score ≥ 900) analysis downloaded from STRING database showed that *DSPP* could establish the connection with MAPK1 and MAPK3 via the interaction of ITGAV, which are important regulatory genes in MAPK signaling pathway (Supplementary Fig. S5c). The relationship between these genes suggested that *DSPP* could affect the efficacy of immunotherapy through MAPK signaling pathway. Estimation of immune-infiltration features showed that *DSPP-*mutant tumors had remarkably higher M1/M2 ratio of macrophage (*P* < 0.001; Fig. 4e) than wild-type tumors. Moreover, *DSPP* mutation was associated with an increased IFN-γ signature gene expression in both our cohort (average score: 0.12 vs 0.08, *P* = 0.182; Supplementary Fig. S6a) and TCGA NSCLC cohort (average score: 0.49 vs 0.30, *P* < 0.001; Supplementary Fig. S6b).

## Discussion

Currently, PD-1/PD-L1 blockade has become a backbone for the treatment in advanced NSCLC patients without driver mutations.^34,35^ Meanwhile, several studies attempted to evaluate the efficacy of PD-1/PD-L1 blockade monotherapy in NSCLC patients with *EGFR* mutations, either at the frontline setting in PD-L1 high-expression population or at later line setting after the failure of *EGFR*-TKI.^36,37^ All of them led to disappointing results. Combination of *EGFR*-TKIs and PD-1 blockade even resulted in high rate of interstitial pneumonitis and deemed not feasible for further clinical development.^23,24^ Moreover, anti-PD-1/PD-L1 monotherapy was also found to be associated with hyperprogressive disease in *EGFR*-mutant NSCLC.^38^ Therefore, an alternative strategy is urgently needed for patients with *EGFR*-mutant NSCLC who became refractory to *EGFR*-TKIs.

Previously, IMPRESS trial found that continuation of gefitinib plus chemotherapy did not show PFS benefit (5.4 versus 5.4 months) than chemotherapy alone in *EGFR*-mutant patients with advanced NSCLC and progressed from first-line *EGFR*-TKI.^39^ Thus, chemotherapy is the current standard of care in this setting with median PFS of 4–5 months and OS of 16–18 months. The present study is the first prospective study to evaluate the efficacy and safety of PD-1 blockade plus chemotherapy as second-line setting in patients with *EGFR*-mutant advanced NSCLC. We found that toripalimab plus carboplatin and pemetrexed had an ORR of 50% with a median PFS of 7.0 months, and a median OS of 23.5 months, which were superior than historical data of traditional chemotherapy. Consistently, subgroup analysis of patients with *EGFR* mutation from IMPOWER 150 study also demonstrated a superior efficacy of the addition of atezolizumab to bevacizumab and chemotherapy than the standard of care.^40^ Taken together, these findings suggest that the chemo-immunotherapy combination is a promising strategy in previously treated patients with *EGFR*-mutant advanced NSCLC.

The most common adverse events were leukopenia, neutropenia, anemia, elevated AST and ALT, nausea, and thrombocytopenia, which were consistent with the side effect of the similar chemo-immunotherapy combination in NSCLC patients without driver mutations.^40^ Interestingly, combination of *EGFR*-TKIs and PD-1 blockade would result in high rate of interstitial pneumonitis in patients with *EGFR*-mutant advanced NSCLC. However, we did not observe any unexpected AEs in this study. All of the immune-related AEs were consistent with previous studies on toripalimab in this study.^29–31^ These findings were reminiscent of the importance of the sequence of EGFR-TKI and immune-checkpoint inhibitors in this population. Notably, PD-1 blockade monotherapy-related thyroid toxicity was not prominent in this study, which also needs to be validated in a large-cohort study.

Biomarker analysis of PD-L1 expression, TMB level, and CD8 + TIL density showed that none of them could predict the efficacy of toripalimab plus carboplatin and pemetrexed. Patients with PD-L1 high expression had an enhanced ORR of 75.0%. Nevertheless, the ORR could reach to 36.8% in patients of negative PD-L1 expression. Similarly, we did not observe the association of TMB with response rate or survival. Notably, we found that patients with high CD8 + TIL density had an inferior median PFS and OS than those with low CD8 + TIL density, which was reiterated in the immune-infiltration analysis of whole-transcriptome sequencing data. Since tumor microenvironment contains not only CD8 + TILs but also immunosuppressive cells, these findings suggest that unraveling distinct tumor-immune microenvironment features of EGFR-mutant lung cancer^41^ might be helpful to identify the benefit population.

Subsequently, we performed an integrated analysis of whole-exome and transcriptome-sequencing data and found that patients with *DSPP* and *TP53* comutations had significantly prolonged PFS than wild type. Among them, *DSPP* mutation, instead of *TP53* mutation, was more potent to associate with superior PFS. Our finding that the *DSPP* mutation correlated with decreased M2 macrophage infiltration might partly be the possible explanation. As we know, M2 macrophage is immunosuppressive via suppressing the antitumor CD8 + T cells and secreting suppressive chemokine or cytokine. Additionally, our integrated analysis also found that MAPK signaling pathway was significantly downregulated in the *DSPP-*mutant group. Previous reports have shown that MAPK signaling pathway plays an important role in TCR signaling cascade^42^ and co-inhibition of MEK and PD-1/PD-L1 interaction is synergistic and resulted in a durable tumor regression.^43^ Therefore, its downregulation of MAPK signaling pathway might also attribute the superior efficacy in the patients with *DSPP* mutation, and the detailed mechanisms warrant investigation in the future.

We should mention that several limitations existed in this study. First, this is a single-arm phase II trial with a limited number of patients and all of them are Asian. Herein, these results should be cautiously interpreted. Second, only patients who failed from 1^st^/2^nd^ generation of EGFR-TKIs and without T790M mutation were included. Therefore, the current findings could not be generalized to the population who had T790M mutation or received first-line osimertinib. Third, the primary endpoint was assessed by investigators in this study; therefore, the absence of central blinded review of ORR might have some bias. Last but not least, it has been reported that the PFS benefits with *EGFR*-TKI plus chemotherapy or anti-angiogenesis in the first-line setting. Therefore, these regimens might challenge the clinical relevance of the present study.

In summary, this phase-II study first provides evidence for the safety, efficacy, and potential predictive biomarkers of the combination of toripalimab with carboplatin and pemetrexed as second-line setting in patients with EGFR-mutant NSCLC, which will be further validated by the ongoing randomized phase-III trial (NCT03924050).

## Patients and methods

### Study design

This study was a multicenter, open-label, phase-II clinical trial evaluating the safety and clinical activity of toripalimab plus pemetrexed and carboplatin for the treatment of advanced NSCLC with *EGFR*-sensitive mutations after failure of EGFR-TKI therapy (NCT03513666). The study was approved by the institutional review board of participating centers and was conducted in accordance with the Declaration of Helsinki and Good Clinical Practice. Each subject provided written informed consent before any procedure of this study.

### Patients’ eligibility

Eligible subjects should be aged 18–75 years old with histologically and/or cytologically confirmed advanced or recurrent NSCLC with *EGFR*-sensitive mutations, failed from prior first-line EGFR-TKI therapy (including gefitinib, erlotinib, icotinib, afatinib, or dacomitinib), did not harbor secondary *EGFR* T790M mutation, have at least one measurable lesion per response-evaluation criteria in solid tumors version 1.1 (RECIST v1.1), with eastern cooperative oncology group (ECOG) performance status of 0 or 1, and adequate organ and bone marrow function. Patients with previously treated asymptomatic brain metastasis were also allowed to enroll into this study. Exclusion criteria included mixed with small-cell lung-cancer component or squamous-cell carcinoma, other driver mutations with available targeting drugs, prior systemic chemotherapy, any active autoimmune disease or a history of autoimmune diseases, prior long-term systemic immunosuppressive therapy, or PD-1/PD-L1 blockade.

### Treatment and endpoints

Two different doses (240 mg or 360 mg) of toripalimab were used for the initial safety and efficacy analysis in 10 patients and then chose one in the subsequent enrolled patients. Patients received toripalimab intravenously once every three weeks until PD, or intolerable side effects. During the induction phase, patients received up to six cycles of 500 mg/m^2^ pemetrexed plus AUC 5 carboplatin via intravenous infusion, once every three weeks. During the maintenance phase, patients received toripalimab plus 500 mg/m^2^ pemetrexed. The washout period between the end of EGFR-TKI and initiation of toripalimab was more than 28 days. Response was assessed every six weeks according to RECIST v1.1 locally by investigators. Patients who initially developed PD were allowed to stay on the study if the investigator considered that patients could benefit from the treatment. Treatment beyond the second PD was not allowed. Adverse events were assessed according to the national cancer institute common terminology criteria for adverse events, version 4.03, and monitored throughout and for 60 days after treatment discontinuation. The primary endpoint was ORR. The secondary end points included safety, DCR, PFS, and OS.

### *EGFR* mutation detection

Tumor rebiopsy samples that have a conformed pathological diagnosis of NSCLC were collected for *EGFR*-mutation testing. *EGFR* (exons 18–22) was sequenced by using genomic DNA. Cycle sequencing of the purified polymerase chain reaction (PCR) products was carried out with PCR primers using the commercially available ADx Mutation Detection Kits (Amoy Diagnostics Company Ltd., Xiamen, China).

### PD-L1 expression

Tumor rebiopsy sample was obtained from each patient before the initiation of this study to perform exploratory PD-L1 expression analysis. PD-L1 expression was detected by immunohistochemistry (IHC) staining with JS311 antibody using ventana benchmark autostainer. PD-L1-positive status was defined as the presence of membrane staining of any intensity in ≥1% of tumor cells. We adopted two different cutoffs of PD-L1 ≥ 1% or PD-L1 ≥ 10% to perform the biomarker analysis.

### CD8^+^TIL density analysis

CD8^+^TIL density was assessed by using a mouse anti-CD8 monoclonal antibody (M7103, clone C8144B, DAKO). Lymphocytes with immunostained CD8 infiltrating within tumor region (central or marginal) were defined as CD8^+^TILs. On the basis of the percentage of CD8^+^TILs displayed within tumor region, we determined high/low CD8^+^TIL density (CD8^+^TIL^+/−^) with cutoff of 5%, which was analogous to the previous studies.^44–46^

### WES and TMB analysis

WES was performed with SureSelect Human All Exon V6 kit (Agilent) on tumor biopsies and matched peripheral blood mononuclear cell (PBMC) samples. The detailed sequencing and analysis process were summarized in Supplemental Materials. Genomic alterations, including single-base substitution (SNV), short and long insertions/deletions (INDELs), copy number variants (CNV), and gene rearrangement and fusions, were assessed. The TMB was determined by analyzing somatic mutations, including coding-base substitution and INDELs per megabase (Mb).

### Whole transcriptome sequencing

Total RNA was extracted from available tumor tissue samples and RNA quality and quantity were determined by capillary electrophoresis on Eukaryote Total RNA Pico chips (Agilent Technologies). The prepared libraries were sequenced on an Illumina HiSeq 2000 sequencer. The details bioinformatic analysis, including identification of DEGs, functional enrichment analyses, immune-infiltration estimation, SVM, and SSN, was summarized in Supplemental Materials.

### Sample size determination and statistical analysis

This is a phase-II trial with a single-stage design. At one-sided significance level of 0.05, a total of 39 patients could provide 80% power to show the efficacy of toripalimab plus chemotherapy in the second-line setting with a target ORR of 50% versus 30% of standard chemotherapy using Clopper–Pearson method.

Safety and efficacy analyses included all patients who received ≥1 dose of study medication. ORR and its 95% exact CI were determined by Clopper and Pearson methodology. PFS and OS were plotted using the Kaplan–Meier method, with median and the corresponding two-sided 95% CIs reported. Duration of response was analyzed with Kaplan–Meier method with data from all responders. The data cutoff for analysis was October 22, 2020. Statistics analyses were performed with SAS or GraphPad Prism software.

## Supplementary information

Supplementary Materials

## Data Availability

The data that support the findings of this study are available from the corresponding author upon reasonable request. Public data resources: The TCGA datasets, including COAD and READ, were downloaded from cBioPortal (http://www.cbioportal.org/).
