# Supplementary material for: Toripalimab plus chemotherapy as second-line treatment in previously EGFR-TKI treated patients with EGFR-mutant-advanced NSCLC: a multicenter phase-II trial
Source: Signal Transduct Target Ther. 2021 Oct 15;6:355. doi: 10.1038/s41392-021-00751-9 (PMC8517012; doi:10.1038/s41392-021-00751-9)
Supplement: Supplementary file 1 — Supplementary Materials [file 41392_2021_751_MOESM1_ESM.docx]

Supplementary Materials for

Toripalimab plus chemotherapy as second-line treatment in previously EGFR-TKIs treated patients with EGFR-mutant advanced NSCLC: a multi-center phase II trial

Tao Jiang^1, 13^, Pingyang Wang^2, 13^, Jie Zhang^1, 13^, Yanqiu Zhao^3^, Jianying Zhou^4^, Yun Fan^5^, Yongqian Shu^6^, Xiaoqing Liu^7^, Helong Zhang^8^, Jianxing He^9^, Guanghui Gao^1^, Xiaoqian Mu^3^, Zhang Bao^4^, Yanjun Xu^5^, Renhua Guo^6^, Hong Wang^7^, Lin Deng^8^, Ningqiang Ma^8^, Yalei Zhang^9^, Hui Feng^10^, Sheng Yao^10^, Jiarui Wu^2^, Luonan Chen^1, 2, 11, 12, *^, Caicun Zhou^1, *^ and Shengxiang Ren ^1, *^

Correspondence to: [caicunzhou_dr@163.com](mailto:caicunzhou_dr@163.com) (C.Z.), [harry_ren@126.com](mailto:harry_ren@126.com) (S.R.), [lnchen@sibs.ac.cn](mailto:lnchen@sibs.ac.cn) (L.C.)

**This PDF file includes:**

Materials and Methods

Figures. S1 to S6

Tables S1 to S2

References

Materials and Methods

Whole-exome sequencing

DNA libraries were subjected to whole-exome capture with SureSelect Human All Exon V6 kit (Agilent) on tumor biopsies and matched peripheral blood mononuclear cells (PBMC) samples. Human Cot-1 DNA (Life Technologies) and xGen universal blocking oligos (Integrated DNA Technologies) were added as blocking reagents to reduce non-specific hybridization. The capture reaction was performed with NimbleGen SeqCap EZ Hybridization and Wash Kit (Roche) and Dynabeads M-270 (Life Technologies) according to manufacturers’ protocols. The captured samples were sequenced on an Illumina HiSeq X-TEN platform with a paired-end run of 2 × 150bp. The quality of each read was initially verified using the software embedded in the HiSeq X-TEN sequence. A FASTQ file was generated for each tested sample for sequence alignment and converted to a BAM file for further analysis.

WES sequence alignment

We performed the FastQC for quality control on the raw sequence data, Fastp was used for data cleaning.^1^ Next, 34 patients with paired tissue and blood samples were processed and aligned to hg38 using the BWA (v0.7.12), GATK MarkDuplicates (v4.0.9.0) and SAMtools (v1.6).^2-4^ GATK BaseRecalibrator (v4.0.9.0) and GATK ApplyBQSR (v4.0.9.0) were employed for Base Quality Score Recalibration (BQSR), somatic SNV were called using GATK MuTect2 (v4.0.9.0) with paired tissue and blood samples, and then filtered by GATK FilterMutectCalls (v4.0.9.0).^5^

SNVs detection and downstream analysis

GATK MuTect2 pipeline was run with default parameters, all SNVs with “PASS” filter flag were kept for the following evaluation.^6^ ANNOVAR (version 2020-06-08) is performed to functionally annotate detected genetic variants by using table_annovar.pl script.^7^ The R package “maftools” (v2.6.05) was used to convert VCF files into MAF files for subsequent analysis, including drawing oncoplot, predicting genesets associated with survival and comparing differential mutation between two cohorts.^8^ Kaplan‐Meier curve and Cox proportional hazards were performed to characterize the relationship between mutant and WT, calculated by R package “survival” (v3.2-7), then visualized by R package “survminer” (v0.4.8).

Whole transcriptome sequencing

Total RNA was extracted from available tumor tissue samples using the Arcturus Pico Pure RNA Isolation Kit (Life Technologies). RNA quality and quantity were determined by capillary electrophoresis on Eukaryote Total RNA Pico chips (Agilent Technologies). To prepare and amplify cDNA from 500 pg of total RNA, the SMART-Seq v4 Ultra Low Input RNA Kit (Clontech) was used according to manufacturer’s instructions. Amplified cDNA was quality controlled by capillary electrophoresis on a Bioanalyzer using High Sensitivity chips (Agilent Technologies). The prepared libraries were sequenced on an Illumina HiSeq 2000 sequencer.

Raw sequence reads were first aligned to the human genome (hg19) using GSNAP. Then, mapped reads were assigned to human genes based on GRCh37.75 annotation. Uniquely mapped reads were used to quantify gene expression, and differential gene expression evaluation was analyzed by Cuffdiff, a subpackage of Cufflinks (v2.1.1) with Ensembl-annotated genes (version 77). The abundance of transcripts (including mRNAs, pseudogenes, noncoding RNAs, and other predicted RNAs) was calculated and normalized in fragments per kilobase of transcript per million mapped reads (FPKM), as described above, from the raw RNA-seq data and used for Gene Set Enrichment Analysis (GSEA, Broad Institute).

Immune infiltration estimation

Fragments Per Kilobase Million (FPKM) matrix from our trial and TCGA downloaded gene expression data were submit to CIBERSORTx online analysis platform (https://cibersortx.stanford.edu) to estimate the abundances of immune cells respectively, the algorithm was run using the LM22 signature for 100 permutations.^9^ The LM22 signature matrix which defined 22 infiltrating immune cell components, including T cells, B cells, NK cells and macrophages. The differences between PR and non-PR groups in our trial, and between mutant and WT in *EGFR* mutant patients obtained from TCGA were examined by Wilcoxon test.

Identification of differentially expressed genes (DEGs) and Functional Enrichment Analyses

The count-based R package “DEseq2” (v1.30.1) was used to perform DEG analysis by comparing PR and non-PR groups, as well as *DSPP* mutant and WT.^10^ The cut-off criteria of | log2FC| > 2 and adjusted P < 0.05 was used to screen DEGs. The biological functional enrichment of DEGs was indentified by KEGG pathway enrichment analyses by using Metascape online analysis platform (https://metascape.org/) with default parameters.^11^ In the following GSEA analysis, all of the expressed genes data were submitted to GSEA software for evaluating the differentially enriched pathways between *DSPP* mutant and WT, gene set c2.cp.kegg.v6.2.symbols.gmt was used as the reference gene list.^12^ In total 178 gene sets, the top 20 up-regulated and down-regulated gene sets were selected for follow-up analysis.

Selection of classifiers by modified support vector machines (SVM) cross validation

We randomly sampled the patients in PR and non-PR groups, of three samples from each group were included in the test set, and the rest 7 PR patients and 5 non-PR patients were used as the training set. According to the expression of each gene, Wilcoxon test was performed to identify the gene with significant difference (P < 0.05) between two groups, which was defined as differential genes. The sklearn.svm.SVC function (C=1, Kernel=”linear”) of Python module “scikit-learn” was performed to seek for candidate genes with classification ability among input differential genes, the test set used for verifying the classification effect. In the classification test of six test samples, the feature combination that can be correctly classified five times will be retained as a potential marker gene set. Next, we repeated the above-mentioned steps 1000 times. After that, we screened each potential gene combination to find genes appear every time, which suggests these genes have stable and efficient classification ability in our trial. We finally obtained 14 marker genes.

Sample-specific network (SSN) and protein-protein interaction network analysis

SSN method allows us to construct individual-specific networks based on molecular expressions of a single sample, to characterize various human diseases at a network level.^13^ We used protein-protein interaction network (combined score≥900) downloaded from STRING database as background network, to fix the edges that compose the network. Normal samples (n=59) from TCGA-LUAD project were employed to construct a reference network, all edges in the reference network were measured by the Pearson correlation coefficients (PCCs) which calculated by the Python module “SciPy”. Followed by adding each of 18 patients in our trial to the group which employed to construct the reference network, after constructing another network by PCCs, we got new perturbed networks. If the differential PCC (ΔPCC) of an edge between reference network and perturbed network is statistically significant (ΔPCC > 0.8 and adjusted P < 0.05), the edge would be kept on the SSN. Statistical analysis was performed using a two-tailed Student's t-test.


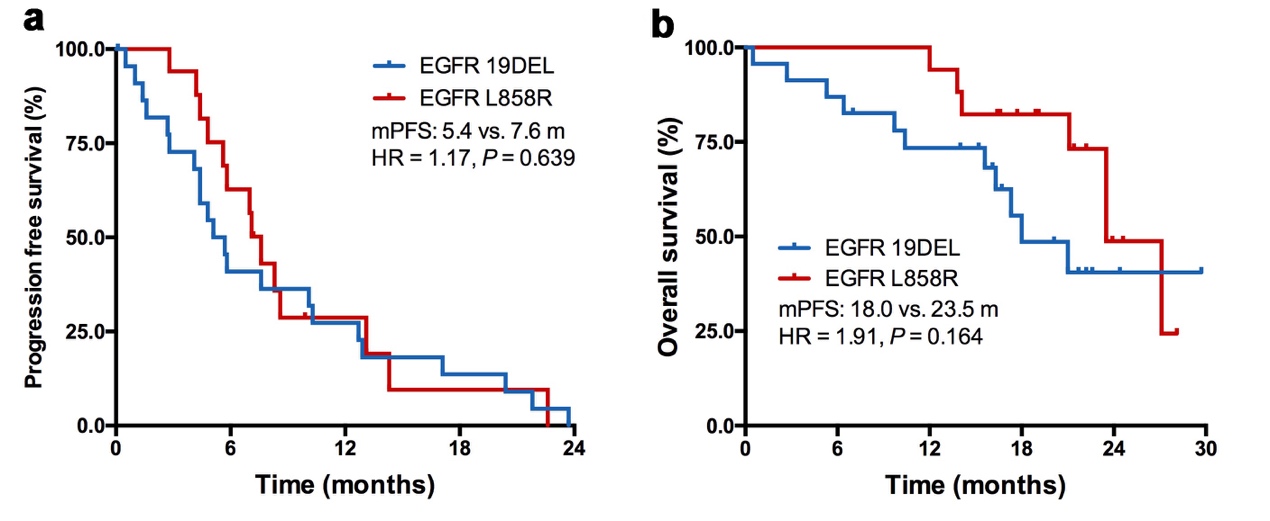


Supplemental Figure. S1.

**Kaplan-Meier curves of survival according to mutation subtypes.** (a) Progression-free survival of patients with *EGFR* exon 19 deletion versus exon 21 L858R mutation (log-rank test). (b) Overall survival of patients with *EGFR* exon 19 deletion versus exon 21 L858R mutation (log-rank test). *EGFR* 19DEL, *EGFR* exon 19 deletion; *EGFR* L858R, *EGFR* exon 21 L858R mutation.


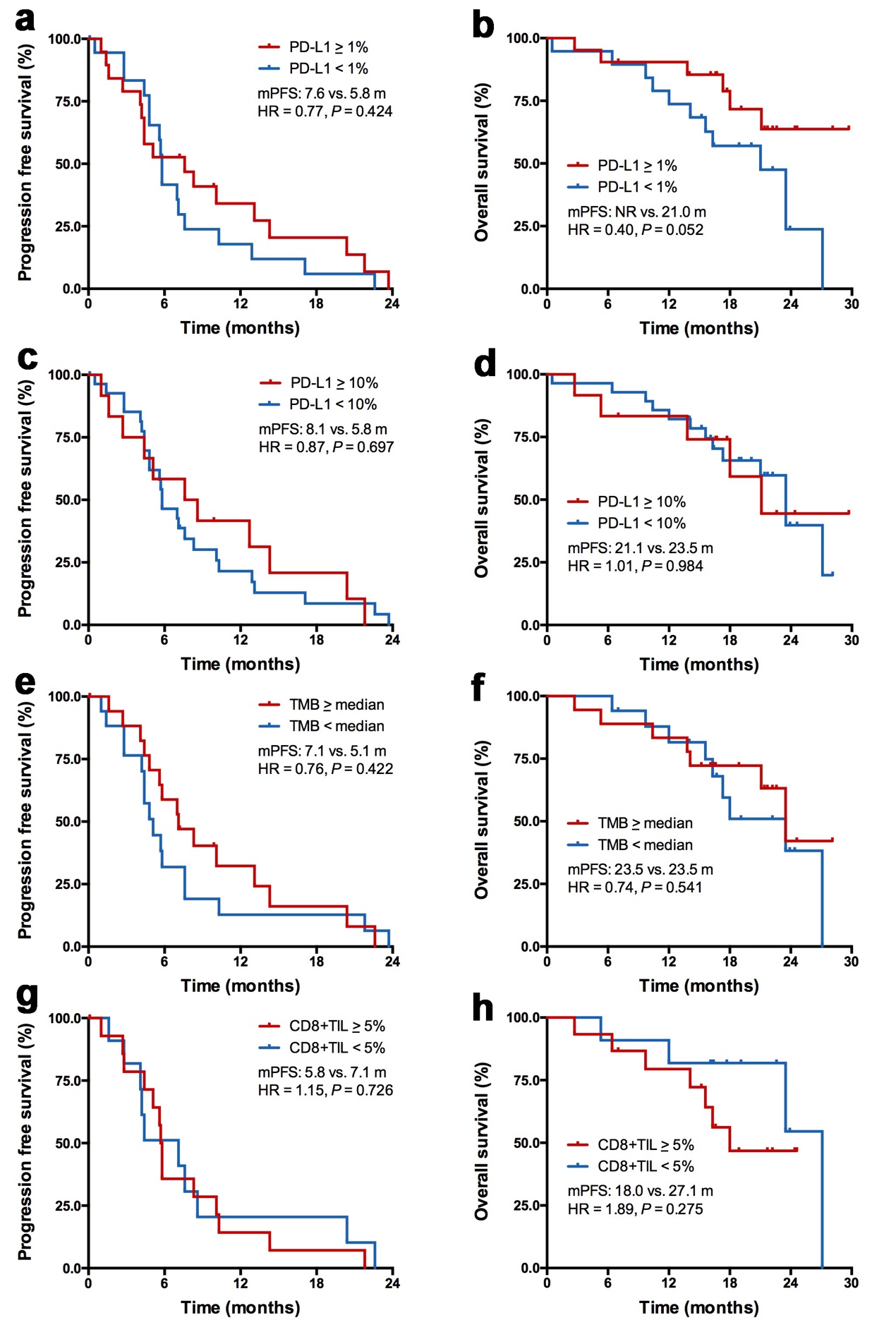


Supplemental Figure. S2.

**Kaplan-Meier curves of survival according to PD-L1 expression, TMB level and CD8+ TILs density.** (a) Progression-free survival of patients with PD-L1 expression≥1 versus PD-L1 expression<1 (log-rank test). (b) Overall survival of patients with PD-L1 expression≥1 versus PD-L1 expression<1 (log-rank test). (c) Progression-free survival of patients with PD-L1 expression≥10 versus PD-L1 expression<10 (log-rank test). (d) Overall survival of patients with PD-L1 expression≥10 versus PD-L1 expression<10 (log-rank test). (e) Progression-free survival of patients with high TMB level (≥median) versus low TMB level (<median; log-rank test). (f) Overall survival of patients with high TMB level (≥median) versus low TMB level (<median; log-rank test). (g) Progression-free survival of patients with high CD8+ TILs density (≥5%) versus low CD8+ TILs density (<5%; log-rank test). (h) Overall survival of patients with high CD8+ TILs density (≥5%) versus low CD8+ TILs density (<5%; log-rank test).

Supplemental Figure. S3.

**Predictive value of TP53, DSPP and TP53+DSPP mutations.** (a) Progression-free survival in patients with TP53 mutation versus wild type (log-rank test). (b) Immune infiltration of TP53 mutation versus wild type from EGFR mutant samples obtained from TCGA (Wilcoxon signed rank test). (c) Overall survival in patients with TP53 mutation versus wild type (log-rank test). (d) Overall survival in patients with TP53+DSPP mutation versus wild type (log-rank test). (e) Overall survival in patients with DSPP mutation versus wild type (log-rank test).


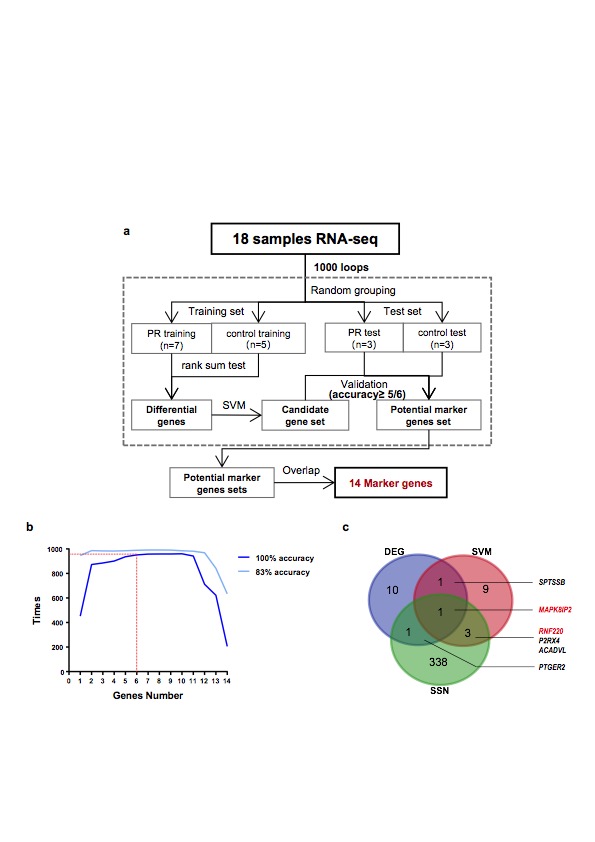


Supplemental Figure. S4.

**Whole transcriptome sequencing. (A)** The flow chart of support vector machines to select PR classifier. **(B)** A line diagram showing classification accuracy. The x-axis shows the number of genes that make up the classifier, and the y-axis shows the amount of times that 1000 loop tests have passed. Two blue lines represent two different accuracy thresholds. The light blue line needs to be correctly classified five times out of six times, while the dark blue line needs to be a monopoly of right. **(C)** Venn graph shows the intersection of candidate genes selected by differential expression analysis, support vector machines and sample-specific network analysis.


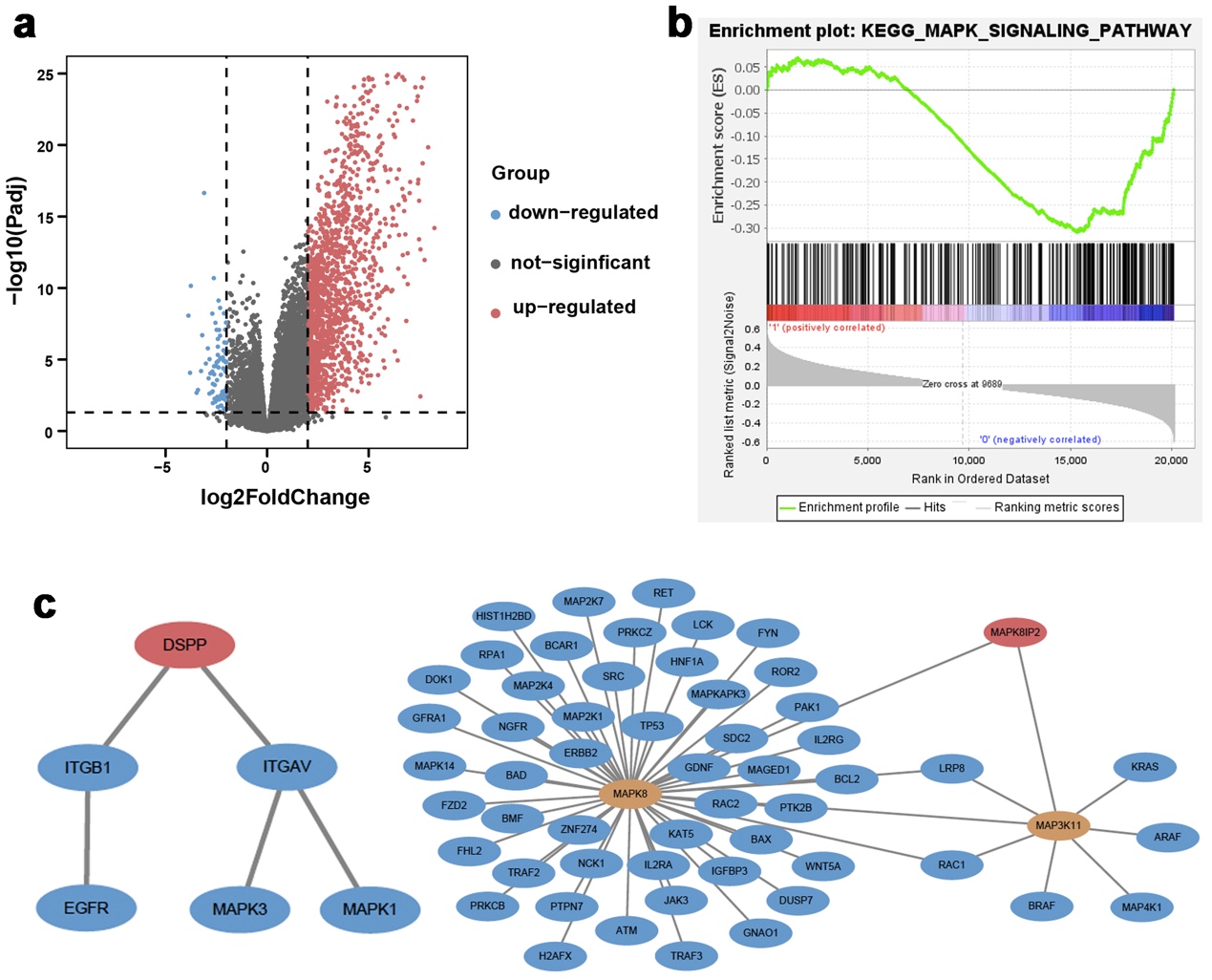


Supplemental Figure. S5.

**Differential expression analysis and protein-protein interaction network between *DSPP*-mutant versus wild-type tumors.** (a) Volcano plot of 1536 up-regulated and 76 down-regulated differentially expressed genes between *DSPP*-mutant versus wild-type tumors from *EGFR-*mutant samples obtained from TCGA (*P* ≤ 0.05, Log_2_Fold Change ≥ 2). (b) GSEA enrichment plot shows down-regulation of MAPK signaling pathway in *DSPP*-mutant versus wild-type tumors (Enrichment Score = -0.309). (c) First-order and key second-order genes linked with DSPP on the background network of PPI, and the local network composed of MAPK8IP2 and its first-order and second-order linked genes on the background network of protein-protein interaction.


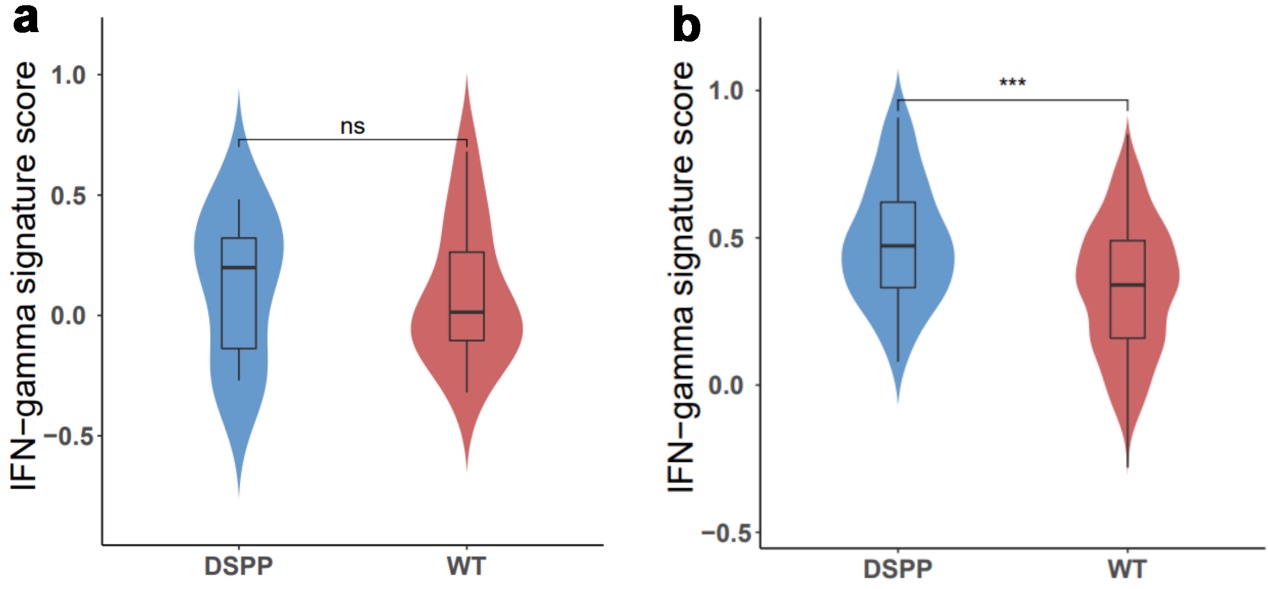


Supplemental Figure. S6.

**Comparison of IFN-γ signature genes expression in both our cohort and TCGA NSCLC cohort.** *DSPP* mutation was associated with an increased IFN-γ signature genes expression in both our cohort **(a)** and TCGA EGFR-mutant NSCLC cohort **(b).** ns, not significant; WT, wild type.

Supplemental Table S1.

Additional biomarker and subgroup analysis for correlation with clinical efficacy.

| **Characteristics** | **Value** | **N** | **ORR (%)** | **95% CI (%)** |
| --- | --- | --- | --- | --- |
| **Age** | **<60** | 23 | 47.8 | 26.8-69.4 |
|  | **≥60** | 17 | 52.9 | 27.8-77.0 |
| **Gender** | **Male** | 19 | 63.2 | 38.4-83.7 |
|  | **Female** | 21 | 38.1 | 18.1-61.6 |
| **Subtype** | **Exon 19 deletion** | 23 | 43.5 | 23.2-65.5 |
|  | **L585R** | 17 | 58.8 | 32.9-81.6 |
| **LDH** | **Normal** | 35 | 54.3 | 36.7-71.2 |
|  | **>ULN** | 5 | 20.0 | 0.005-71.6 |
| **PD-L1*** | **Positive** | 21 | 61.9 | 38.4-81.9 |
|  | **Negative** | 19 | 36.8 | 16.3-61.6 |
| **1^st^ line TKI treatment** | **Gefitinib** | 20 | 35.0 | 15.4-59.2 |
|  | **Icotinib** | 16 | 62.5 | 35.4-84.8 |
|  | **Erlotinib** | 4 | 75.0 | 19.4-99.4 |
| **Total** |  | 40 | 50.0 | 33.8-66.2 |

LDH, Lactate dehydrogenase; ULN, upper limit of normal.

*Positive defined as ≥1% of tumor cells expressing PD-L1 by JS311 IHC staining

Supplemental Table S2.

| Subsequent treatment for the included patients. | |
| --- | --- |
| **Treatment regimens** | **N (%)** |
| 1st EGFR-TKI | 2 (10.0%) |
| 1st EGFR-TKI + Bevacizumab | 1 (5.0%) |
| Dacomitinib | 1 (5.0%) |
| Osimertinib | 3 (15.0%) |
| Chemotherapy | 4 (20.0%) |
| Bevacizumab | 3 (15.0%) |
| Anlotinib | 2 (10.0%) |
| Chemotherapy + Bevacizumab | 4 (20.0%) |
| Local therapy | 1 (5.0%) |

**References**

1 Chen, S., Zhou, Y., Chen, Y. & Gu, J. fastp: an ultra-fast all-in-one FASTQ preprocessor. *Bioinformatics* **34**, i884-i890 (2018).

2 Li, H. *et al.* The Sequence Alignment/Map format and SAMtools. *Bioinformatics* **25**, 2078-2079 (2009).

3 McKenna, A. *et al.* The Genome Analysis Toolkit: a MapReduce framework for analyzing next-generation DNA sequencing data. *Genome Res* **20**, 1297-1303 (2010).

4 DePristo, M. A. *et al.* A framework for variation discovery and genotyping using next-generation DNA sequencing data. *Nat Genet* **43**, 491-498 (2011).

5 Van der Auwera, G. A. *et al.* From FastQ data to high confidence variant calls: the Genome Analysis Toolkit best practices pipeline. *Curr Protoc Bioinformatics* **43**, 11 10 11-11 10 33 (2013).

6 Cibulskis, K. *et al.* Sensitive detection of somatic point mutations in impure and heterogeneous cancer samples. *Nat Biotechnol* **31**, 213-219 (2013).

7 Wang, K., Li, M. & Hakonarson, H. ANNOVAR: functional annotation of genetic variants from high-throughput sequencing data. *Nucleic Acids Res* **38**, e164 (2010).

8 Mayakonda, A., Lin, D. C., Assenov, Y., Plass, C. & Koeffler, H. P. Maftools: efficient and comprehensive analysis of somatic variants in cancer. *Genome Res* **28**, 1747-1756 (2018).

9 Steen, C. B., Liu, C. L., Alizadeh, A. A. & Newman, A. M. Profiling Cell Type Abundance and Expression in Bulk Tissues with CIBERSORTx. *Methods Mol Biol* **2117**, 135-157 (2020).

10 Love, M. I., Huber, W. & Anders, S. Moderated estimation of fold change and dispersion for RNA-seq data with DESeq2. *Genome Biol* **15**, 550 (2014).

11 Zhou, Y. *et al.* Metascape provides a biologist-oriented resource for the analysis of systems-level datasets. *Nat Commun* **10**, 1523 (2019).

12 Subramanian, A. *et al.* Gene set enrichment analysis: a knowledge-based approach for interpreting genome-wide expression profiles. *Proc Natl Acad Sci U S A* **102**, 15545-15550 (2005).

13 Liu, X., Wang, Y., Ji, H., Aihara, K. & Chen, L. Personalized characterization of diseases using sample-specific networks. *Nucleic Acids Res* **44**, e164 (2016).
